# Experience with postmortem computed tomography in the forensic analysis of the November 2015 Paris attacks

**DOI:** 10.1080/20961790.2020.1802686

**Published:** 2020-11-02

**Authors:** Laura W. de Jong, Laurence Legrand, Tania Delabarde, Ghazi Hmeydia, Myriam Edjlali, Lilia Hamza, Joseph Benzakoun, Catherine Oppenheim, Bertrand Ludes, Jean-François Meder

**Affiliations:** a Department of Neuroradiology, GHU Paris Psychiatrie et Neurosciences - Sainte-Anne Hospital, Université de Paris, Paris, France; b Inserm U1266, IMA-Brain, Institut de Psychiatrie et Neurosciences de Paris, Paris, France; c Pôle Universitaire d’Imagerie Post-Mortem, Université de Paris, Paris, France; d Institut Médico-Légal de Paris, Paris, France; e Service d’Accueil des Urgences, Hôpital Avicenne, Bobigny, France; f Université de Paris, BABEL, CNRS, Paris, France

Several reports discuss the added value of postmortem computed tomography (PMCT) in the forensic analysis of mass fatality incidents (MFIs), such as the 2009 Victorian bushfires, the Grenfell tower disaster and the MH17 plane crash [1–5]. In France, the first deployment of PMCT in an MFI took place during the forensic analysis of the November 2015 Paris terrorist attacks. On 13 November 2015, attacks with automatic firearms and suicide vests ended the lives of 130 people at multiple sites in Paris, namely around the football stadium (the Stade de France), at restaurant bar terraces and in the Bataclan concert theatre (Figure 1), and led to many more injured who were admitted to multiple hospitals. These terrorist attacks were classified as open disasters; i.e. disasters that resulted in the death and injury of many hundreds for whom no prior records or descriptive data were available that could help identify potential victims [6]. The criminal trial of the alleged assailants in the Paris attacks is expected to begin in 2020. To avoid potential interference with the ongoing preparatory judicial inquiry, it is currently prohibited to publish the results of the PMCT findings of the victims. However, considering that experience with PMCT in the forensic analysis of open disasters was still limited in 2015, we recount the organisation of scanning the corpses and share the lessons learnt from the Paris attacks to raise further awareness of the value of PMCT in the forensic analysis of MFIs and its potential valuable contribution to the disaster victim identification (DVI) chain.

**Figure 1.**
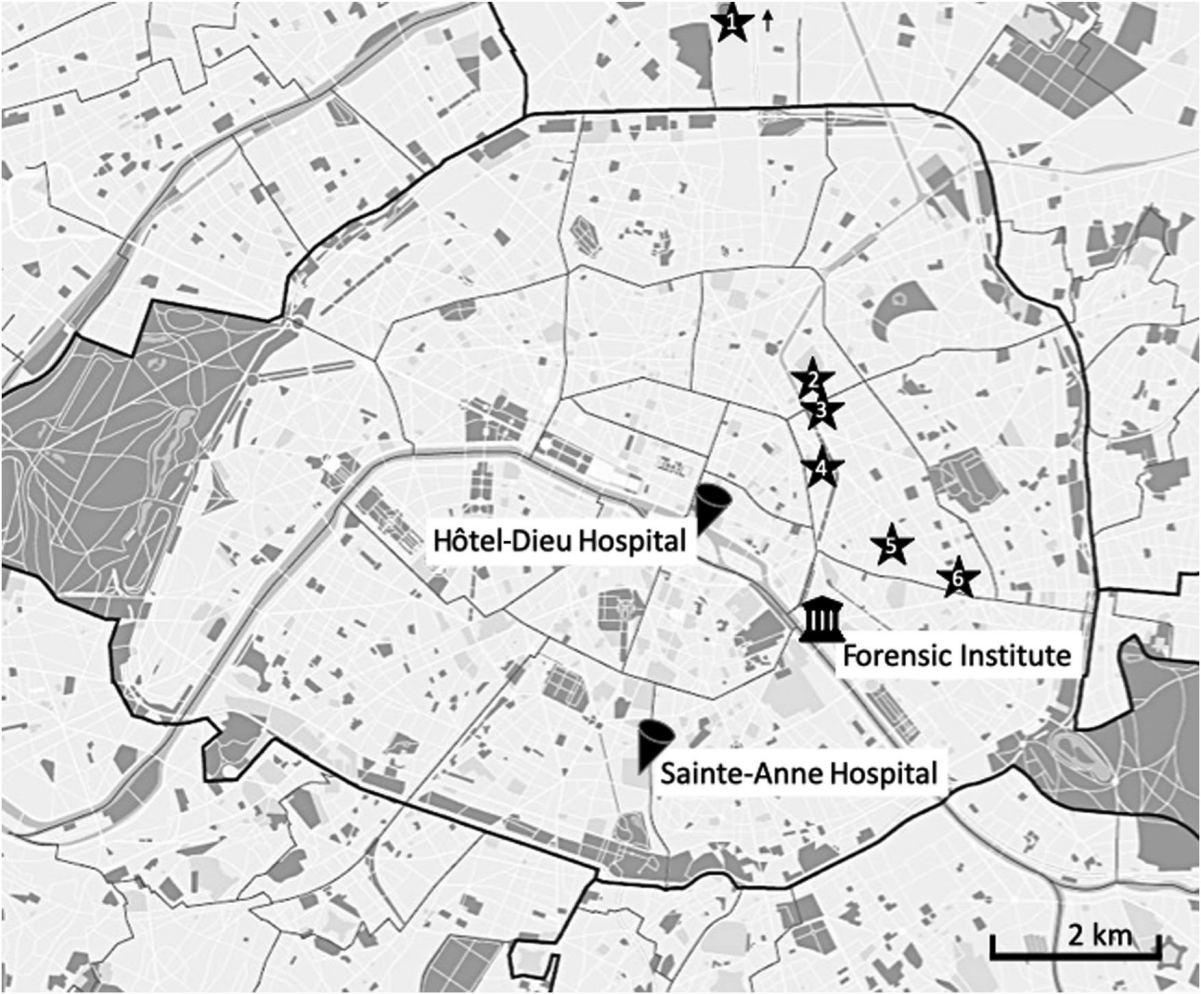
Map of the area affected in the Paris attacks and the locations of the hospitals and the Forensic Institute that were involved in the investigation. 1. Stade de France, Saint-Denis; 2. Rue Alibert et Rue Bichat; 3. Rue de la Fontaine au Roi; 4. Bataclan theatre; 5. Rue de Charonne; 6. Boulevard Voltaire.

## Organisation of the forensic analysis

The Central Directorate of the Judicial Police (DCJP) was in charge of the investigation of the November attacks, which was supervised by the prosecutor of Paris. The difficulties that were faced in the identification process of the victims and the adoption of INTERPOL’s DVI protocol [7,8] are described elsewhere [9]. Because PMCT was not deployed as part of the identification process at the time, these findings will not be recounted here. The forensic pathologic analysis was conducted by the forensic team, which originated from the Forensic Institute in Paris, and it began almost instantly after the arrival of the first bodies. The team consisted of 15 pathologists (10 from Paris, 1 from Lille, 1 from Strasbourg and 3 from the Institut de Recherche Criminelle Gendarmerie Nationale (IRCGN)) as well as 4 odontologists, 4 radiographers, 6 radiologists, 1 anthropologist, 3 ballistics experts (IRCGN) and 22 autopsy technicians. The team was located at the Forensic Institute in Paris, except for the radiologists and radiographers, who were located at the Sainte-Anne Hospital and Hôtel-Dieu Hospital. Focusing on the victims only, a total of 130 whole bodies and many more body parts were analysed. The prosecutor, advised by the forensic team, ordered PMCT scanning for a large number of the corpses to facilitate (1) localisation of bullets and other projectiles as evidence for the types of weapons used and (2) the descriptions of the injuries.

**Figure 2.**
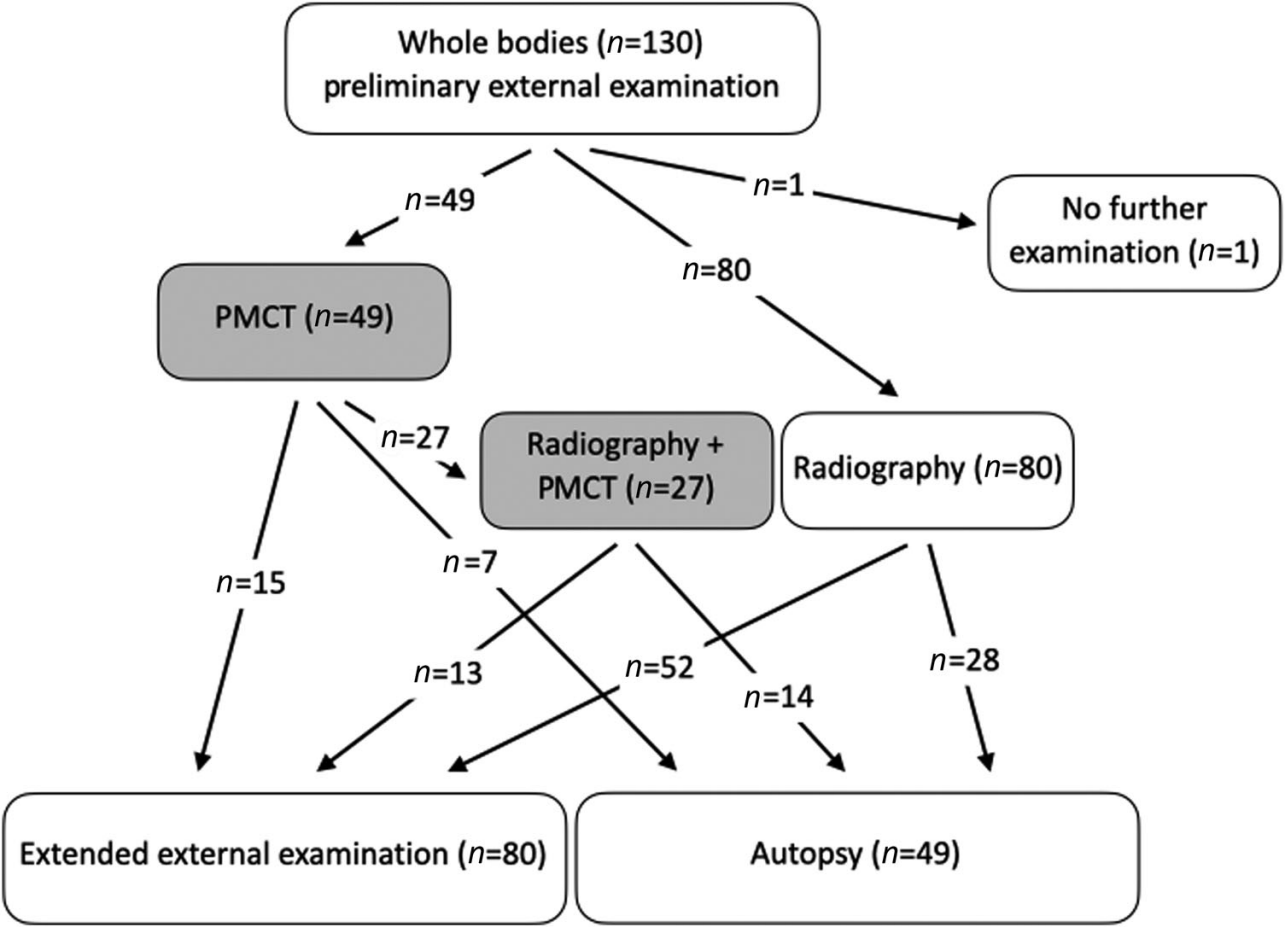
Number of forensic operations performed from 14 to 19 November 2015. *Extended external examination with minimal invasive opening of the body for the purpose of collecting bullets. PMCT: postmortem computed tomography.

Preliminary external examination, radiography and PMCT took place before autopsy upon the body’s entry to the Forensic Institute. Radiographs were performed at the Forensic Institute by autopsy assistants on a Prestilix 1600 (General Electric Medical Systems, Milwaukee, WI, USA) for direct use by the forensic pathologists to localise radio-opaque foreign bodies, and they were not reviewed by the radiologists. There was no standardisation in the views, number of views or which anatomical areas were radiographed, but most examinations involved imaging the whole body. Although the aim was to scan as many of the bodies as possible, the total number of PMCT examinations that could be performed in hospital settings was limited by the availability of the scanner. The following factors were considered important but not exclusive when selecting the corpses that were scanned: corpses with clear fatal singular injuries (usually those to the head or neck) on preliminary external examination were not scanned; corpses with multiple injuries to the legs and arms or for whom it was unclear which injury was fatal were scanned. Two radiological centres with expertise in PMCT analysis were deployed. Over a period of 6 d after the attacks, 49 corpses were scanned, 39 at the Sainte-Anne Hospital and 10 at the Hôtel-Dieu Hospital. The results of the PMCT performed at Hôtel-Dieu Hospital were sent directly to the prosecutor and cannot be accessed under current rules; therefore, these results are not part of the scope of this letter. After scanning, 28 cases underwent extended external examination with minimal invasive opening of the body to collect ballistic elements. The remaining 21 underwent autopsy. Figure 2 displays the numbers and types of forensic operations in the first days after the attacks in November 2015.

## PMCT acquisition and protocol

**Figure 3.**
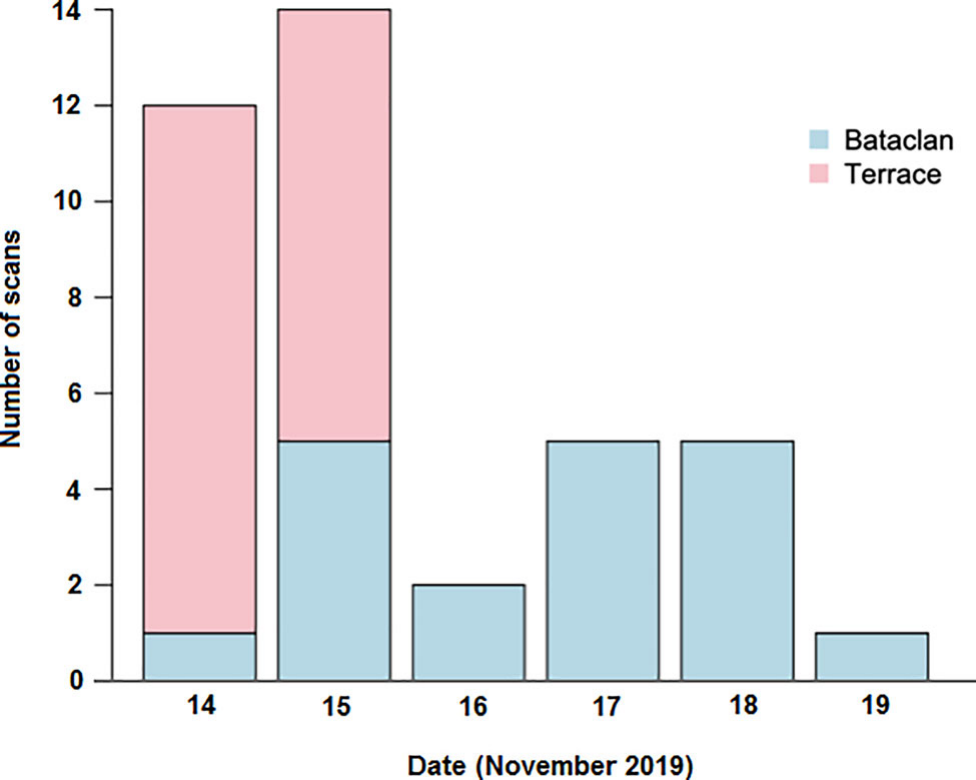
Time scale of the postmortem computed tomography (PMCT) scanning at Sainte-Anne Hospital.

On the morning of Saturday, 14 November 2015, an area of the Sainte-Anne Hospital was isolated from the public by a team from the national police. A blue tent was placed on the hospital grounds close to the entrance of the CT scanning suite to receive the incoming corpses and prepare them for scanning. The clinical departments of the hospital were alerted, and the hospital’s normal scanning programme was cancelled except for emergency cases. A dedicated image reading room was made available and was located next to the scanner, separate from other activities in the radiological department and accessed only by members of the forensic team. The room was shielded from the public and other hospital personnel, had no windows and did not border corridors used by patients. Four scanner technicians, all with previous experience with PMCT, volunteered and came to the hospital for scanner image acquisition. Four radiologists with ≥ 1 year of expertise performing PMCT volunteered to perform the image analysis. Transportation and scanning of corpses occurred between the 14th and 19th of November. Up to six bodies at the same time were transported to the Sainte-Anne Hospital by funeral convoys. Figure 3 shows the number of PMCT scans per day acquired at the Sainte-Anne Hospital. Generally, corpses of those who had died on the terraces were scanned earlier than those who died in and around the Bataclan theatre. This was because it took longer for the latter to be transported to the forensic institute because of the ongoing crime scene investigation at the theatre. Only whole corpses were scanned.

The corpses were scanned using a 64-detector CT scanner (GE Lightspeed; General Electric Medical Systems, Milwaukee, WI, USA) in closed body bags to maintain hygiene and to respect the victims’ anonymity. The corpses were transported from the tent to the scanner by personnel from the funeral transport and placed on the CT table with help from and instruction by the CT technicians. The protocol included anteroposterior and lateral scout views; two main spiral scans (one focused on the head and neck [120 kV, 250 mA (with automated dose modulation), 0.8 s/rotation, pitch of 0.531, beam collimation 0.625 × 64, slice thickness 2.5 mm, slice interval 1.25 mm and display field of view 32 cm]; another focused on the chest, abdomen, pelvis and limbs [120 kV, 250 mA (dose modulation), 0.7 s/rotation, pitch of 1.375, beam collimation 0.625 × 64, slice thickness 1.25 mm, slice interval 1.25 mm and display field of view 50 cm]); and supplemental spiral scans for the limbs, if required. Data were transferred to a dedicated GE Advantage Windows post-treatment workstation (version 4.6) for multiplane and 3 D volume rendering reconstructions. The following images were stored long-term on a separate and encrypted drive accessible only by the forensic radiologist team: 1.25 mm slices of the head, neck and arms reconstructed with soft tissue and bone kernels; 1.25 mm slices of the chest-abdomen-pelvis reconstructed with soft tissue kernels and 2.50 mm slices with bone kernel; 2.50 mm slices of the legs reconstructed in soft tissue and bone kernels. Post-reconstructions were made by the radiologists.

## Image analysis

**Figure 4.**
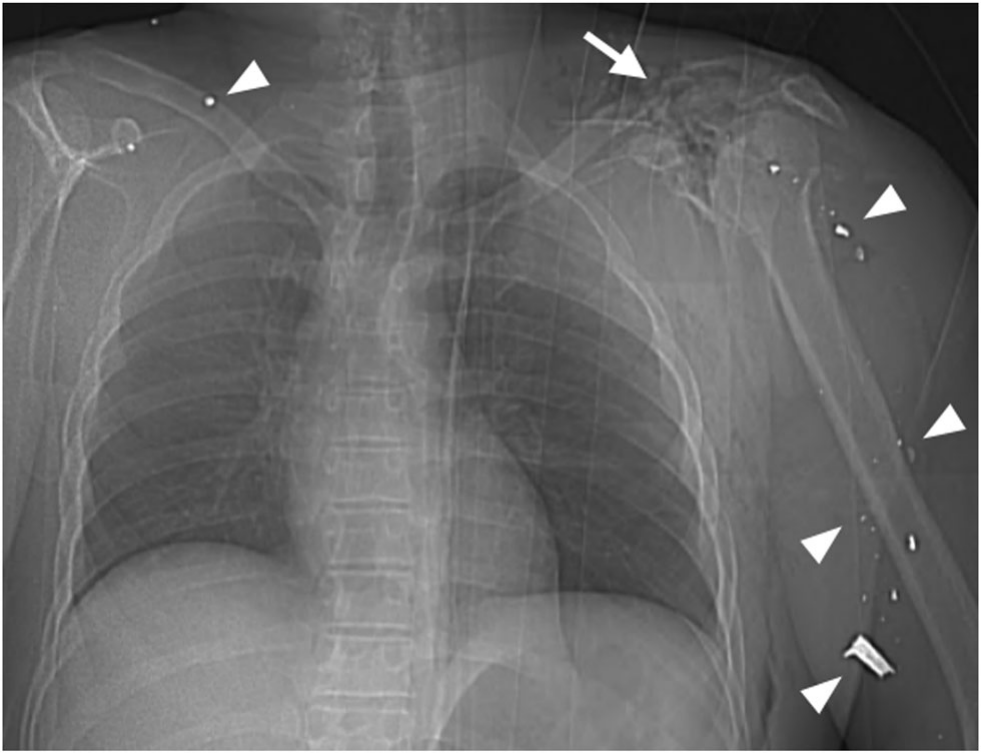
Part of a topogram at chest level showing multiple ballistic fragments (arrowheads) in the soft tissues of the right lower neck and the left upper arm. Note the comminuted fracture of the left shoulder (arrow). Lungs and mediastinum seemed unaffected. The victim died of severe ballistic head injury (not shown).

**Figure 5.**
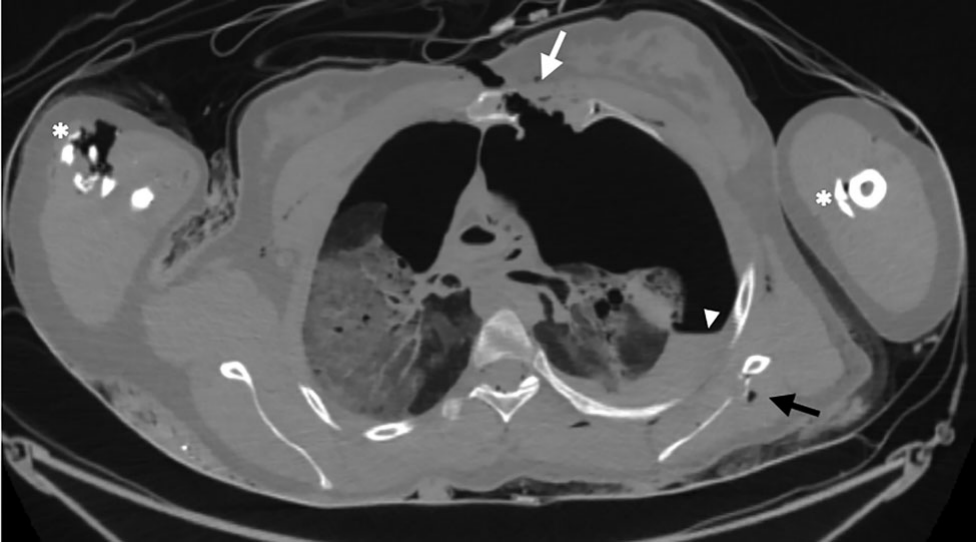
Axial postmortem computed tomography (PMCT) image at the level of the chest showing a clear intrathoracic ballistic trajectory with an anterior entry hole and diastasis of the presternal skin and subcutaneous fat. Note the perforation of the sternum anteriorly (white arrow) and the perforation of the left scapula posteriorly (black arrow) with outward-directed bone fragments indicative of the direction of the projectile. Left pneumothorax with a hyperdense liquid is visible posteriorly (arrowhead), indicative of haemorrhage in the thoracic cavity. There are also fractures of the left and right humerus (asterisks).

**Figure 6.**
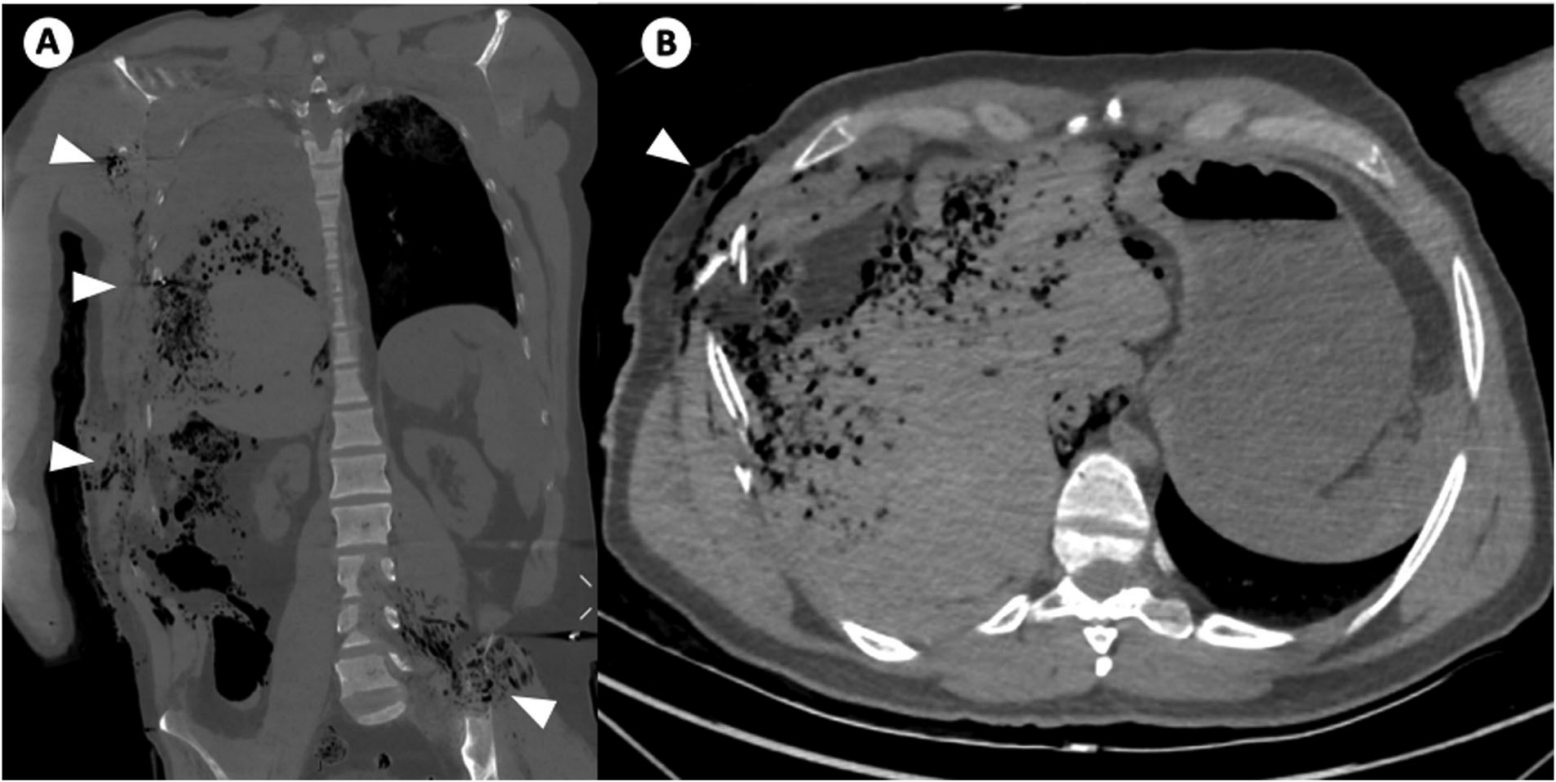
Coronal postmortem computed tomography (PMCT) image of the chest and abdomen (A) and axial PMCT image at the level of the upper abdomen (B) of a victim with multiple ballistic impact sites (arrowheads). Partially overlapping and dispersing trajectories complicate the identification of different ballistic trajectories.

Each scan was read by two radiologists. Findings from the preliminary external examination were available before image analysis. During image analysis, a forensic pathologist from the forensic institute was present in the room to record the presence and location of intra- or extra-corporeal projectiles and the presence of potentially relevant personal items in the body bags (such as telephones, wallets, jewelery, etc.) as determined by the radiological team. Scans were read in a standardised fashion; reports were typed by hand (no speech recognition or picture archiving and communication system was used) and each followed the same structure. First, the topogram was screened for obvious metallic foreign bodies or projectiles, metallic fragment clouds, presence and type of bone fractures and other post-traumatic injuries (such as (haemo-)pneumothorax, (haemo-)pneumocephalus and (haemo-)pneumoperitoneum). Figure 4 displays part of a topogram showing multiple ballistic fragments. Subsequently, the axial thin slices were analysed per body region (head and face, neck, thorax, abdomen and pelvis and limbs), and visceral and bone lesions were described. Clear separate projectile trajectories leaving an almost linear trace in organs and adjacent bones were noted (Figure 5). However, because of the nature of the ammunition used in the attacks, multiple impacts on the body often gave rise to dispersing and overlapping post-traumatic injuries (Figure 6). Therefore, the account of projectile trajectories in the radiology reports likely underestimated the actual number of perforating trajectories. The conclusion of the report stated the presence and locations of projectiles, a description of the major impact sites and potential lethal injuries. All PMCT scans were interpreted immediately after acquisition.

## Integration of the results

Preliminary reports were printed twice, and images were burned on a CD and transported to the Forensic Institute by a member of the forensic team within hours of acquisition. Key images from the reports and native scan images were available during the autopsy. Final reports containing the requisition of the prosecutor, a certificate of the service performed and oath taking were sent by certified mail with return receipt to the judiciary parquets. The forensic teams used the description of where bullets and/or other projectiles were located to guide extended external examination to collect the material. The radiological findings, especially of the description of potential lethal injuries, were integrated in the final forensic pathology report, and a reconciliation meeting was held on 23 November 2015.

## Lessons learnt

During the debriefing sessions in the months following the attacks, the role of PMCT as part of the analysis was reviewed, and several lessons were drawn. First, PMCT reports provided detailed descriptions of the locations and types of injury, covering the entire body. This information assisted the forensic pathologist, after integrating all information, to determine potentially fatal injuries and estimate the post-injury survival time of the victims. This was considered particularly important because of the delay of more than 2.5 h between the beginning of the attacks at the Bataclan theatre and the final intervention by the special units of the national police. During this time, no medical aid could be provided to the victims dying in the theatre. Second, PMCT provided information that supported the ballistic analysis. PMCT also enabled rapid determination of the presence and precise location of bullets and other metallic projectiles, either intracorporeally or inside the body bag. Overall, in 15/28 cases, PMCT provided sufficient evidence, and the forensic team refrained from autopsy. As a result, the entire investigation was faster, as was the return of the bodies to the families of the deceased. Considering that, on average, 12 autopsies were performed per day on three different autopsy tables (a fourth autopsy table was reserved for INTERPOL and the investigation of body fragments), our team estimated that the use of PMCT in only 37.7% of victims shortened the investigation by 1.25 to 2.33 d.

Furthermore, in the months after November 2015, other long-term advantages of the use of PMCT in MFIs became apparent. PMCT images can be re-analysed at any point in time and thus, can be reviewed without the stress of the moment and can add a significant amount of data initially missed. PMCT can also be used to answer unanticipated questions or results can be sent to international or remote experts if further analyses are warranted. The possibility of long-term storage of the PMCT images proved useful. In the months after the attacks, rumours of mutilation/castration and even beheading of the victims in the Bataclan theatre began circulating on social media [10]. However, PMCT provided evidence that no such mutilations were found in the bodies that were scanned.

The debriefing sessions also formulated guidelines for future PMCT deployment in MFIs. The first guideline was that PMCT should be an integrated part of the forensic investigation and be acquired for all bodies and body parts of victims of MFIs. Second, PMCT should be obtained during the first phase of the DVI chain. The acquisition of PMCT does not depend on external examination and does not need to be delayed. This guideline was also motivated by the recognition of the tremendous potential carried by PMCT to assist the DVI chain. When it comes to primary identifiers (i.e. DNA, friction ridge analysis, comparative dental analysis) adding a dental scan to the PMCT protocol can expedite the work of the forensic odontologist in the postmortem unit [11]. However, using dental scans in MFIs is potentially limited by overheating of the X-ray tube, which can cause it to fail prematurely. Therefore, the decision to use dental scans depends on multiple factors, namely the available scanner capacity, the number of casualties, intactness of the face and the primary goal of the PMCT (identification *vs*. lesion analysis). When recording secondary identifiers (e.g. personal description, tattoos, property, clothing items found on the body prosthesis, medical findings, pacemakers and other implants), PMCT has great potential. In collaboration with the forensic anthropologist, PMCT can produce a biological profile, including estimated biological age, sex, ancestry and stature. Finally, PMCT is an excellent tool for identification by comparison with postmortem and antemortem scans, if available.

In 2018, the International Society for Forensic Radiology and Imaging (ISFRI) published a statement on the role of PMCT in DVI [12], and in 2019, INTERPOL adopted the radiology reporting form into the pink (postmortem) forms [6]; however, this was not yet a part of the DVI protocol at the time of the attacks in 2015. The November 2015 Paris attacks can be marked as a transitional period in France regarding the use of PMCT in the forensic investigation of MFIs. Indeed, the lessons learnt were well incorporated in the forensic investigation of the terrorist attack of Nice on 14 July 2016 [13]. For that MFI, all bodies and body parts were scanned, and the PMCT results accelerated the DVI chain by providing information on body measurements, secondary identifiers (scars, anatomical variants, stigmata of previous surgery, etc.) and personal items that were found within the body bag (jewelery, clothes). Furthermore, following international trends, the forensic institute obtained a CT scanner entirely dedicated to PMCT in 2018 to expand its capacity to deploy PMCT not only for individual cases but also for future MFIs.

## References

[1] Cordner SM, Woodford N, Bassed R. Forensic aspects of the 2009 Victorian bushfires disaster. Forensic Sci Int. 2011;205:2–7. 20832958 10.1016/j.forsciint.2010.08.008

[2] O’Donnell C, Iino M, Mansharan K, et al. Contribution of postmortem multidetector CT scanning to identification of the deceased in a mass disaster: experience gained from the 2009 Victorian bushfires. Forensic Sci Int. 2011;205:15–28. 20691550 10.1016/j.forsciint.2010.05.026

[3] Rutty GN, Biggs MJP, Brough A, et al. Remote post-mortem radiology reporting in disaster victim identification: experience gained in the 2017 Grenfell Tower disaster. Int J Legal Med. 2020;134:637–643. 31250083 10.1007/s00414-019-02109-x PMC7044252

[4] Khoo LS, Hasmi AH, Abdul Ghani Aziz SA, et al. MH17: the Malaysian experience. Malays J Pathol. 2016;38:1–10. 27126658

[5] Sidler M, Jackowski C, Dirnhofer R, et al. Use of multislice computed tomography in disaster victim identification—advantages and limitations. Forensic Sci Int. 2007;169:2–3. 10.1016/j.forsciint.2006.08.004 16997522

[6] INTERPOL. Disaster victim identification guide. Lyon (France): INTERPOL; 2018. Available from: https://www.interpol.int/en/content/download/589/file/18Y1344EDVI_Guide.pdf

[7] Sweet D. INTERPOL DVI best-practice standards—an overview. Forensic Sci Int. 2010;201:18–21. 20303223 10.1016/j.forsciint.2010.02.031

[8] Kvaal SI. Collection of post mortem data: DVI protocols and quality assurance. Forensic Sci Int. 2006;159:S12–S14. 16574361 10.1016/j.forsciint.2006.02.003

[9] de Boer HH, Roberts J, Delabarde T, et al. Disaster victim identification operations with fragmented, burnt, or commingled remains: experience-based recommendations. Forensic Sci Res. 2020;5:191–201. 33224550 10.1080/20961790.2020.1751385 PMC7654639

[10] Libération [Internet]. Paris. 2019. Available from: https://www.liberation.fr/checknews/2017/11/14/bonjour-y-a-t-il-eu-des-tortures-au-bataclan_1652742S.C. French.

[11] Toupenay S, Cheikh AB, Ludes B, et al. Forensic odontology identification response to terrorist attacks in Paris November 2015. Forensic Sci Res. 2020;5:214–222. 33209505 10.1080/20961790.2020.1778847 PMC7646575

[12] Shelmerdine SC, Hutchinson JC, Al-Sarraj S, et al. British Neuropathological Society and International Society of Forensic Radiology and Imaging expert consensus statement for post mortem neurological imaging. Neuropathol Appl Neurobiol. 2018;44:663–672. 29533475 10.1111/nan.12482

[13] Quatrehomme G, Toupenay S, Delabarde T, et al. Forensic answers to the 14th of July 2016 terrorist attack in Nice. Int J Legal Med. 2019;133:277–287. 29666997 10.1007/s00414-018-1833-5

